# Genome-scale CRISPR screening for modifiers of cellular LDL uptake

**DOI:** 10.1371/journal.pgen.1009285

**Published:** 2021-01-29

**Authors:** Brian T. Emmer, Emily J. Sherman, Paul J. Lascuna, Sarah E. Graham, Cristen J. Willer, David Ginsburg

**Affiliations:** 1 Department of Internal Medicine, University of Michigan, Ann Arbor, Michigan, United States of America; 2 Life Sciences Institute, University of Michigan, Ann Arbor, Michigan, United States of America; 3 Chemical Biology Program, University of Michigan, Ann Arbor, Michigan, United States of America; 4 Department of Computational Medicine and Bioinformatics, University of Michigan, Ann Arbor, Michigan, United States of America; 5 Department of Human Genetics, University of Michigan, Ann Arbor, Michigan, United States of America; 6 Department of Pediatrics and Communicable Diseases, University of Michigan, Ann Arbor, Michigan, United States of America; 7 Howard Hughes Medical Institute, University of Michigan, Ann Arbor, Michigan, United States of America; University of California, Los Angeles, UNITED STATES

## Abstract

Hypercholesterolemia is a causal and modifiable risk factor for atherosclerotic cardiovascular disease. A critical pathway regulating cholesterol homeostasis involves the receptor-mediated endocytosis of low-density lipoproteins into hepatocytes, mediated by the LDL receptor. We applied genome-scale CRISPR screening to query the genetic determinants of cellular LDL uptake in HuH7 cells cultured under either lipoprotein-rich or lipoprotein-starved conditions. Candidate LDL uptake regulators were validated through the synthesis and secondary screening of a customized library of gRNA at greater depth of coverage. This secondary screen yielded significantly improved performance relative to the primary genome-wide screen, with better discrimination of internal positive controls, no identification of negative controls, and improved concordance between screen hits at both the gene and gRNA level. We then applied our customized gRNA library to orthogonal screens that tested for the specificity of each candidate regulator for LDL versus transferrin endocytosis, the presence or absence of genetic epistasis with *LDLR* deletion, the impact of each perturbation on LDLR expression and trafficking, and the generalizability of LDL uptake modifiers across multiple cell types. These findings identified several previously unrecognized genes with putative roles in LDL uptake and suggest mechanisms for their functional interaction with LDLR.

## Author summary

The level of cholesterol circulating in the blood in low-density lipoproteins (LDL) is an important determinant of overall risk for cardiovascular diseases, including heart attack and stroke. This level is regulated by the removal of LDL from circulation into liver cells. While many molecules involved in LDL uptake have been characterized, we hypothesized that other currently unrecognized genetic interactions are also involved in this process. We therefore applied CRISPR-mediated genome editing to systematically test the contribution of every gene in the human genome to the uptake of LDL by a liver-derived cell line. We synthesized a secondary CRISPR library targeting the top candidate genes from this initial genome-wide screen to confirm their role in LDL uptake and to test their influence on other cellular functions. Our findings confirm the role of genes previously known to participate in LDL uptake and also provide novel insight into the overall regulation of this process.

## Introduction

Atherosclerotic cardiovascular disease is the leading cause of morbidity and mortality worldwide. A preponderance of evidence from epidemiology, human genetics, animal studies, and clinical trials have established that dysregulation of cholesterol homeostasis is a key factor in the pathogenesis of atherosclerosis[1]. Cholesterol is transported in the bloodstream in the form of lipoproteins, lipid-protein complexes that are typically characterized by their buoyancy during fractionation by ultracentrifugation. Cholesterol circulating in low-density lipoprotein (LDL) and other apolipoprotein B-containing lipoproteins exhibits a particularly strong correlation with atherosclerosis, and therapies that lower LDL cholesterol reduce the rate of cardiovascular disease. LDL cholesterol levels are tightly controlled through the complex interplay between intestinal absorption of dietary cholesterol, *de novo* cholesterol biosynthesis, efflux of cholesterol from peripheral tissues, and cellular uptake of lipoproteins[2].

A rich history of discovery in diverse fields including genetics, cell biology, and biochemistry has elucidated many of the molecular determinants of LDL regulation[3–5]. LDL is cleared from circulation by the LDL receptor (LDLR). The extracellular domain of LDLR directly binds to the apolipoprotein B component of LDL particles, triggering the receptor-mediated endocytosis of the LDLR-LDL complex into clathrin-coated vesicles. Internalized complexes of LDL and LDLR traffic through the endolysosomal pathway until luminal acidification triggers their dissociation, with cholesterol being extracted from LDL while LDLR either recycles back to the cell surface or, if bound to its negative regulator PCSK9, traffics to lysosomes for degradation. The importance of LDL uptake to human cholesterol regulation and cardiovascular disease is highlighted by the monogenic causes of familial hypercholesterolemia that affect this pathway[6], including mutations in the genes encoding for LDLR itself, its ligand apolipoprotein B, its negative regulator PCSK9, or its endocytic adapter LDLRAP1. An additional level of regulation of the genes involved in cholesterol uptake and synthesis is provided by SREBP signaling, in which low cellular sterol levels lead to the SCAP-mediated trafficking of SREBP proteins from the ER to the Golgi, where they are cleaved by resident proteases (encoded by *MBTPS1* and *MBTPS2*) to release and activate their transcription factor domains[7,8]. Human genome-wide association studies (GWAS) have also identified >250 loci associated with blood lipid levels[9–11]. Despite these many successes, our molecular understanding of LDL regulation remains incomplete. For the majority of GWAS associations, the causal link to a specific gene and the mechanism for the genotype-phenotype correlation remains unknown. Moreover, only an estimated 20–30% of the heritability of lipid traits is currently explained[12]. It is therefore likely that additional, as yet unrecognized genetic interactions contribute to cholesterol regulation in humans.

Recent advances in genome editing and massively parallel DNA sequencing have enabled high-throughput functional interrogation of the mammalian genome[13]. We previously performed a genome-wide CRISPR screen for the molecular determinants of PCSK9 secretion, leading to our identification of SURF4 as a cargo receptor that recruits PCSK9 into the secretory pathway[14]. We now report adaptation of this approach to screen for modifiers of cellular LDL uptake. Through a primary genome-wide CRISPR screen, followed by the synthesis and re-screening of a focused secondary gRNA library with greater depth of coverage, we validated 118 positive regulators and 45 negative regulators of HuH7 cell LDL uptake. Orthogonal screening, in which this customized guide RNA (gRNA) library was applied to other phenotypic selections, enabled further characterization of putative hits for their specificity in influencing the endocytosis of LDL, the nature of their interaction with LDLR, and their generalizability across cell types.

## Results

### Primary genome-scale CRISPR screen for modifiers of HuH7 cell LDL uptake

HuH7 cells, originally derived from a well-differentiated human hepatocellular carcinoma [15], are widely used as a model of hepatocyte LDL uptake and exhibit functional regulatory networks of cholesterol regulation and uptake, SREBP regulation, and statin sensitivity[16]. We first investigated the time- and dose-dependence of LDL uptake by HuH7 cells. LDL uptake by HuH7 cells was readily detectable above cellular autofluorescence by flow cytometry following a 1 hour incubation with 4 μg/mL of fluorescently-conjugated LDL in serum-free media (S1A Fig). These conditions were in the linear range of detection with respect to both LDL dose and duration of incubation (S1B and S1C Fig). Acquisition of fluorescent signal was mediated by LDLR, as CRISPR-mediated targeting of *LDLR* resulted in a ~75% reduction in LDL uptake under these conditions (S1D Fig). Pre-incubation of HuH7 cells with lipoprotein-depleted media resulted in an *LDLR*-dependent ~67% increase in LDL uptake, consistent with upregulation of *LDLR* expression via SREBP signaling (S1D Fig). These results suggest that this model system recapitulates the LDLR-dependence and SREBP-responsiveness of cellular LDL uptake and is a suitable platform for genome-wide screening.

**Fig 1:**
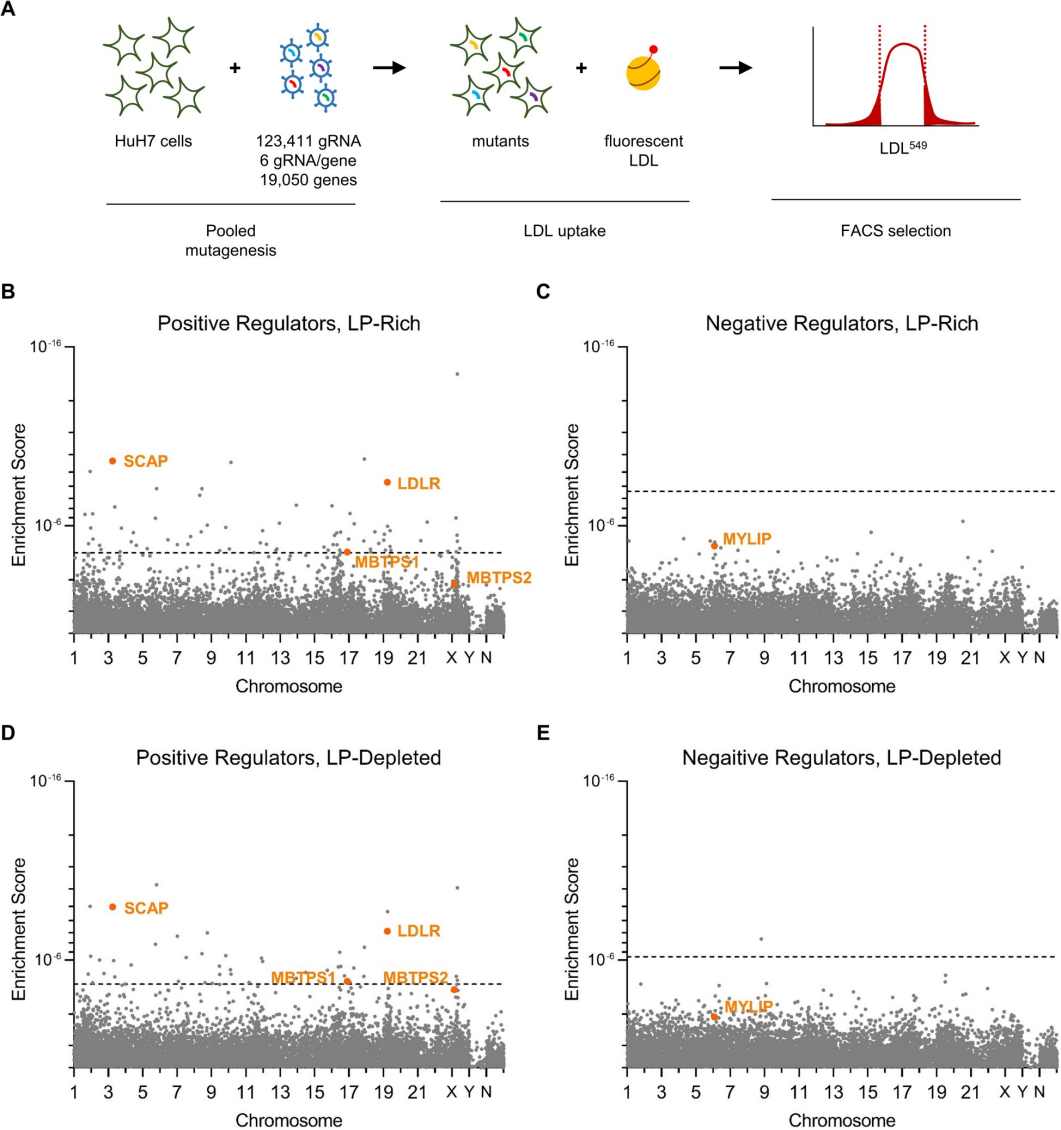
Primary genome-wide CRISPR screens for HuH7 LDL uptake modifiers. (A) Schematic of screening strategy. (B-E) MAGeCK gene level enrichment scores for genes whose perturbation causes reduced LDL uptake (B, D) or increased LDL uptake (C, E) under lipoprotein-rich (B-C) or lipoprotein-depleted (D-E) culture conditions.

To comprehensively identify genetic modifiers of HuH7 cell LDL uptake, we transduced ~25 million cells with the pooled GeCKOv2 lentiviral library delivering Cas9 and 123,411 gRNAs, including 6 gRNA for nearly all known protein-coding genes in the genome[17] (Fig 1A). Following 13 days of expansion in culture, to facilitate target site mutagenesis and turnover of wild-type protein, cells were split and cultured for an additional 1 day, either under continued lipoprotein-rich or changed to lipoprotein-depleted growth conditions. Mutant cells were then incubated with fluorescently-conjugated LDL and sorted by flow cytometry into bins of LDL^high^ (top 7.5%) and LDL^low^ (bottom 7.5%) cells. Massively parallel sequencing of amplified gRNA sequences from each bin was performed and the relative enrichment of each gRNA in either pool was assessed. A total of 3 independent biologic replicates were performed for each screen.

Gene-level analysis identified 95 candidate genes with a false discovery rate (FDR) <5% whose targeting was associated with reduced LDL uptake under either lipoprotein-rich or lipoprotein-deficient conditions (Figs 1B, 1C and S1, S2 Tables). Among these candidates were known regulators of LDL uptake including *LDLR*, *SCAP*, and *MBTPS1*, though *MBTPS2* was not identified among the screen hits (ranking 343 and 58 under lipoprotein-rich and lipoprotein-depleted conditions, with FDR>5% for both). A high degree of concordance was observed for identified positive regulators between the screens conducted under lipoprotein-rich or lipoprotein-depleted conditions, with 27/95 hits identified under both conditions (S2 Fig). Genes positively identified under lipoprotein-rich conditions were 246-fold more likely than negative genes to also be identified under lipoprotein-depleted conditions; genes positively identified under lipoprotein-depleted conditions were 384-fold more likely than negative genes to also be identified under lipoprotein-rich conditions.

Only 1 gene, *SQLE*, was identified whose targeting was associated with enhanced LDL uptake (Fig 1D and 1E and S1, S2 Tables). The positive control *MYLIP*, encoding the LDLR negative regulator IDOL[18], was ranked 7 and 138 among negative regulators under lipoprotein-rich and lipoprotein-depleted conditions but did not meet the FDR<5% threshold for genome-wide significance. The identification of several positive controls and the concordance of our hits across screening conditions suggested that our primary screen was successful, though limited by background noise at genome scale.

### Secondary screen validation of HuH7 LDL uptake modifiers

**Fig 2:**
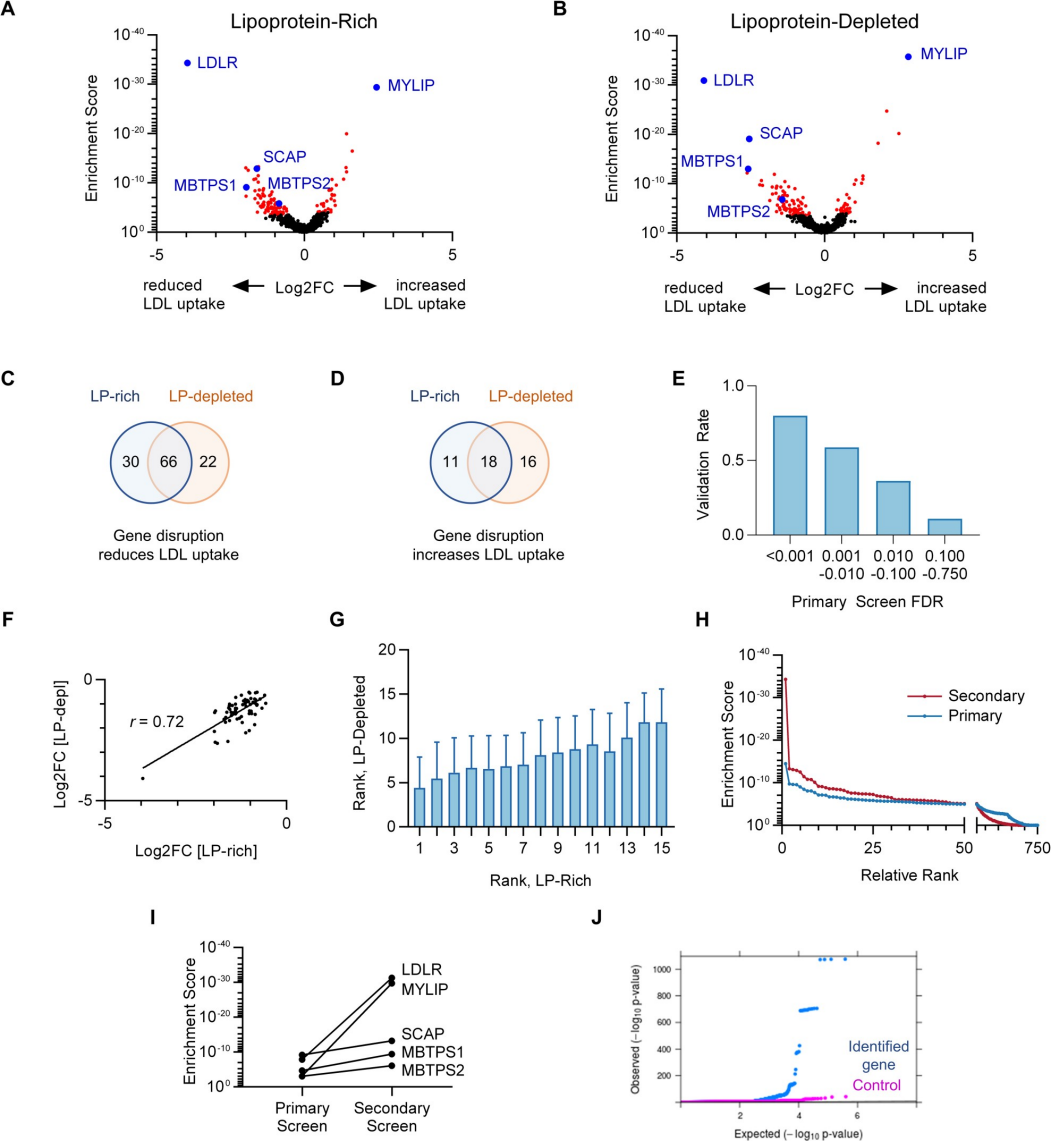
Targeted secondary CRISPR screens for modifiers of LDL uptake by HuH7 cells. (A-B) Volcano plots displaying MAGeCK gene level enrichment scores and associated gRNA log2 fold changes for each gene tested in the secondary gRNA library, under lipoprotein-rich (A) or lipoprotein-depleted (B) cultured conditions, with genes identified with FDR<5% displayed in red and positive controls in blue. (C-D) Venn diagrams of genes identified whose targeting was associated with reduced (C) or enhanced (D) cellular LDL uptake under lipoprotein-rich and/or lipoprotein-depleted culture conditions. (E) Genes identified in the primary screen for LDL uptake were stratified by FDR tier and compared for their validation rate (FDR<5%) in the secondary screen for LDL uptake. (F) Correlation of effect size for genes identified as positive regulators of LDL uptake under both lipoprotein-rich and lipoprotein-depleted culture conditions. (G) Average relative ranking of each individual gRNA among the 15 gRNA per gene in lipoprotein-depleted conditions relative to ranking of that same gRNA in lipoprotein-rich conditions. (H) Cumulative distribution function of MAGeCK enrichment scores for genes tested in both the primary and secondary CRISPR screens for LDL uptake. (I) Comparison of MAGeCK gene level enrichment scores for positive control genes in the primary versus secondary CRISPR screens for LDL uptake. (J) QQ plot of LDL GWAS results in UK Biobank within identified LDL uptake regulator genes compared to matched control genes.

To validate and refine our primary screen hits, we next developed a focused secondary screen of candidate genes with greater depth of coverage. We applied our validation testing to an extended list of potential LDL uptake regulators (positive regulators FDR<50%, negative regulators FDR<75%), reasoning that false negatives might lie further down our candidate list due to a variety of factors including inadequate gRNA efficiency or depth of coverage in the primary screen. We designed and synthesized a custom CRISPR library containing 12,207 gRNAs, including 15 gRNA per gene for 554 potential positive regulators and 170 potential negative regulators, along with 1000 control non-targeting sequences. Massively parallel sequencing of the plasmid pool of this library confirmed the presence of 99.97% of gRNA sequences inserted into the CRISPR plasmid backbone, (S3A Fig) with minimal library skewing (S3B Fig). We generated lentiviral pools from this plasmid mix and performed a secondary screen for HuH7 cell LDL uptake using conditions that were identical to our primary screen, aside from greater depth of coverage at all stages owing to the smaller library size (S4 Fig). Using an FDR cutoff of 5% in our secondary screen, we identified 118 positive regulators of HuH7 LDL uptake (Fig 2A and 2B and S3 Table), with 66 of these exhibiting significant effects under both lipoprotein-rich and lipoprotein-depleted conditions (Fig 2C). We also identified 45 negative regulators, with 18 of these exhibiting significant effects under both lipoprotein-rich and lipoprotein-depleted conditions (Fig 2D). The validation rate of candidates in the secondary screen was strongly correlated to the strength of signal in the primary screen (Fig 2E). As in the primary screen, genes identified under either lipoprotein-rich or lipoprotein-depleted conditions were much more likely to be identified under the other condition (S5A and S5B Fig), with a high degree of correlation for the relative effect size under either condition (Fig 2F). This concordance between screen conditions also extended to the individual gRNA level, as the relative ranking (Fig 2G) and magnitude of enrichment (S5C Fig) for individual gRNAs under lipoprotein-rich conditions was correlated with their corresponding value under lipoprotein-depleted conditions.

In accordance with its greater depth (both in terms of gRNA per gene tested, and cells per gRNA tested), the secondary screen exhibited more robust performance than the primary screen. More genes were identified with FDR<5%, suggesting an increased power of detection. Screen hits exhibited a clearer discrimination from genes with no signal (Fig 2H). Positive control genes *LDLR*, *SCAP*, and *MBTPS1* were again positively identified in the secondary screen, while *MBTPS2* and *MYLIP* (negative in the primary screen) were readily detected as positive hits in the secondary screen. Each of these internal control genes was identified with more significant enrichment (Fig 2I) and rose in the relative rankings, with *LDLR* and *MYLIP* becoming the top hits for reduced and enhanced LDL uptake, respectively, both under lipoprotein-rich and lipoprotein-depleted culture conditions.

### HuH7 LDL uptake regulators are enriched for LDL GWAS associations

Ontology analysis of our validated HuH7 LDL uptake regulators revealed significant enrichment for several annotations including genes involved in regulation of gene expression, cholesterol metabolism, Golgi to plasma membrane transport, protein N-linked glycosylation, and ubiquitin-mediated protein degradation (S6 Fig and S4 Table). Comparison to current human GWAS data from UK Biobank showed a significant enrichment for genes in proximity to genetic variants associated with LDL cholesterol relative to matched control genes. A total of 163 genes were identified to be either positive or negative regulators of HuH7 LDL uptake. Of these, 12% (20/163) had a genome-wide significant GWAS result (p-value < 5x10^-8^) within the gene while 33% (54/163) had a significant result within 500 kb. P-values for association with LDL cholesterol within the 163 identified genes were also more significant, on average, than those within length-matched control genes (two-sided p-value < 2.2x10^-16^, Fig 2J and S5 Table). The majority of our screen hits had not previously been implicated in cholesterol regulation.

### Most LDL uptake regulators do not cause a similar influence on transferrin uptake

**Fig 3:**
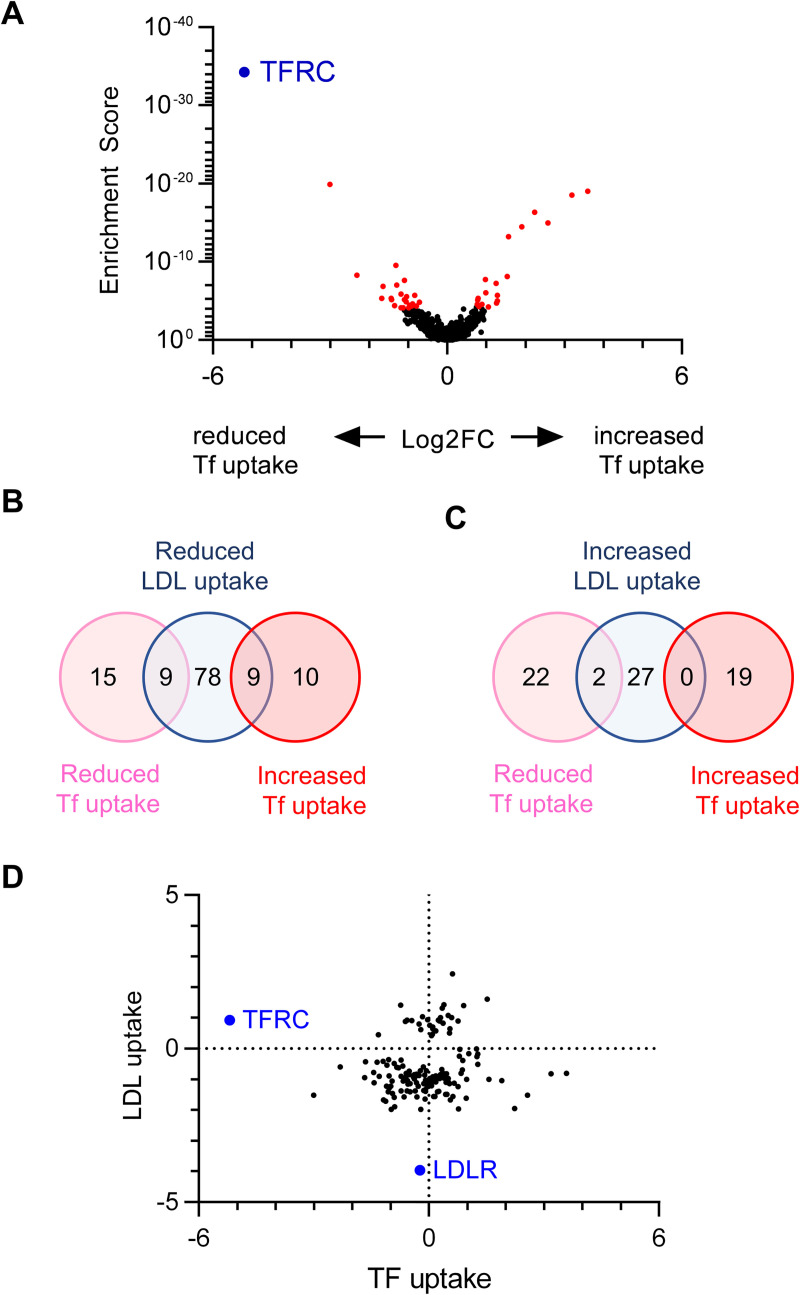
Orthogonal CRISPR screen for modifiers of transferrin uptake by HuH7 cells. (A) Volcano plot displaying transferrin uptake MAGeCK gene level enrichment scores and log2 fold change for each gene tested in the customized gRNA library, with genes identified with FDR<5% displayed in red and *TFRC* in blue. (B-C) Venn diagrams of genes identified whose targeting was associated with reduced (B) or increased (C) cellular transferrin uptake, in comparison to the effect of targeting each gene on HuH7 LDL uptake. (D) Relative effect sizes with log2 fold change for targeting of each gene on transferrin and LDL uptake.

To assess for nonspecific effects on global endocytosis, we next applied our customized gRNA library to assess HuH7 uptake of fluorescently-conjugated transferrin. *TFRC* was included among the secondary library gRNA target genes as a positive control. As expected, *TFRC* was the top hit whose disruption was associated with reduced transferrin uptake (Fig 3A and S6 Table). Among the 736 genes tested with our secondary library, 24 were found to positively regulate and 19 to negatively regulate transferrin uptake (FDR<0.05). Little concordance was observed between regulators of LDL and transferrin uptake (Fig 3B–3D). Surprisingly, disruption of several genes resulted in decreased LDL uptake but enhanced transferrin uptake. Thus, the majority of hits from our secondary screen do not appear to result from global disruption or stimulation of receptor-mediated endocytosis.

### A subset of regulators influence LDL uptake independently of LDLR

**Fig 4:**
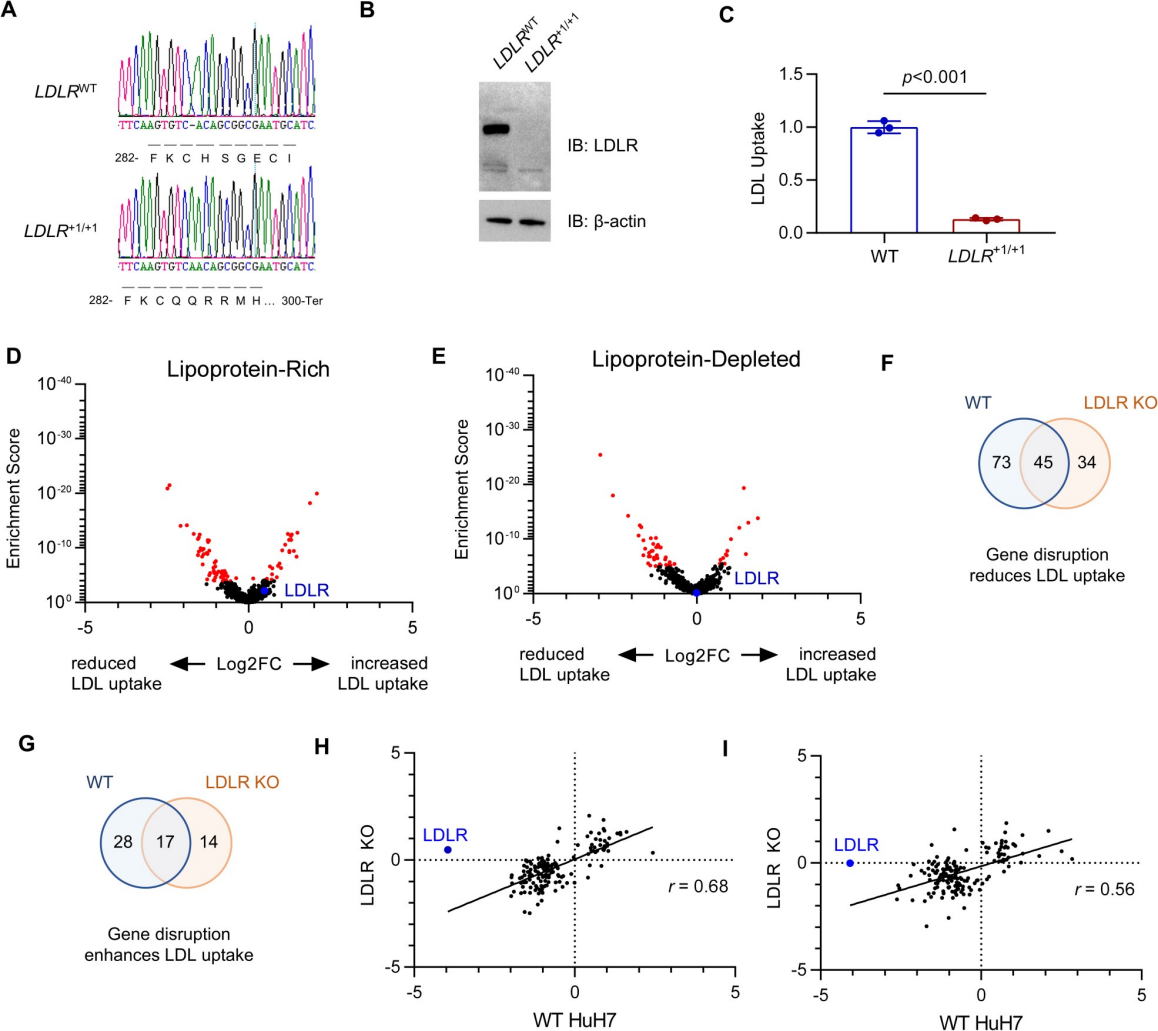
Orthogonal CRISPR screen for modifiers of LDL uptake by *LDLR*-deleted HuH7 cells. (A) Genotyping at the genomic DNA target site, (B) immunoblotting, and (C) quantification of LDL uptake by flow cytometry for a single cell HuH7 clone targeted by CRISPR at the *LDLR* locus. (D-E) Volcano plots displaying MAGeCK gene level enrichment scores and log2 fold change for each gene tested in the secondary gRNA library, under lipoprotein-rich (D) or lipoprotein-depleted (E) cultured conditions, with genes identified with FDR<5% displayed in red. (F-G) Venn diagrams demonstrating the overlap in genes identified from HuH7 WT and *LDLR* KO cells for genes whose disruption was associated with reduced (F) or enhanced (G) LDL uptake. (H-I) Comparison of effect size on LDL uptake in WT and *LDLR* KO cells under lipoprotein-rich (H) or lipoprotein-depleted (I) conditions for each gene showing a significant effect in either background.

Since the majority of fluorescent LDL acquisition under our screening conditions was *LDLR*-dependent (S1D Fig), we hypothesized that most of our screen hits would influence LDL uptake via interaction with LDLR, and therefore would exhibit no effect on LDL uptake when tested on a *LDLR*-deleted genetic background. To test this hypothesis, we generated an HuH7 clone harboring a homozygous frameshift mutation in *LDLR* (Fig 4A), with no detectable LDLR protein by immunoblotting (Fig 4B) and a ~85% reduction in LDL uptake relative to parental wild-type cells (Fig 4C). We then screened this *LDLR*-deleted cell line with our secondary CRISPR library under lipoprotein-rich and lipoprotein-depleted culture conditions (Fig 4D and 4E and S7 Table). Surprisingly, we found that many modifiers of LDL uptake identified in wild-type cells were also identified in *LDLR*-deleted cells, with 45/118 positive and 17/45 negative regulators exhibiting similar effects on LDL uptake in *LDLR*-deleted cells (Fig 4F–4I). The majority of LDLR-independent LDL uptake regulators (44/79 positive regulators, 18/31 negative regulators) were identified under both lipoprotein-rich and lipoprotein-depleted conditions (S7A and S7B Fig). Ontology analysis of genes identified under either condition revealed enrichment in multiple annotations including cholesterol biosynthesis, metabolism, and vesicular trafficking (S7C Fig). The identification of these genes is unlikely to be due to an influence on residual *LDLR* expression, as *LDLR*-targeting gRNAs were not enriched in LDL^low^ cells. Instead, these findings suggest that a significant subset of the LDL uptake modifiers identified here may influence LDLR-independent, or both LDLR-dependent and independent, LDL uptake.

### A subset of LDL uptake regulators modulate steady-state LDLR expression and trafficking to the cell surface

**Fig 5:**
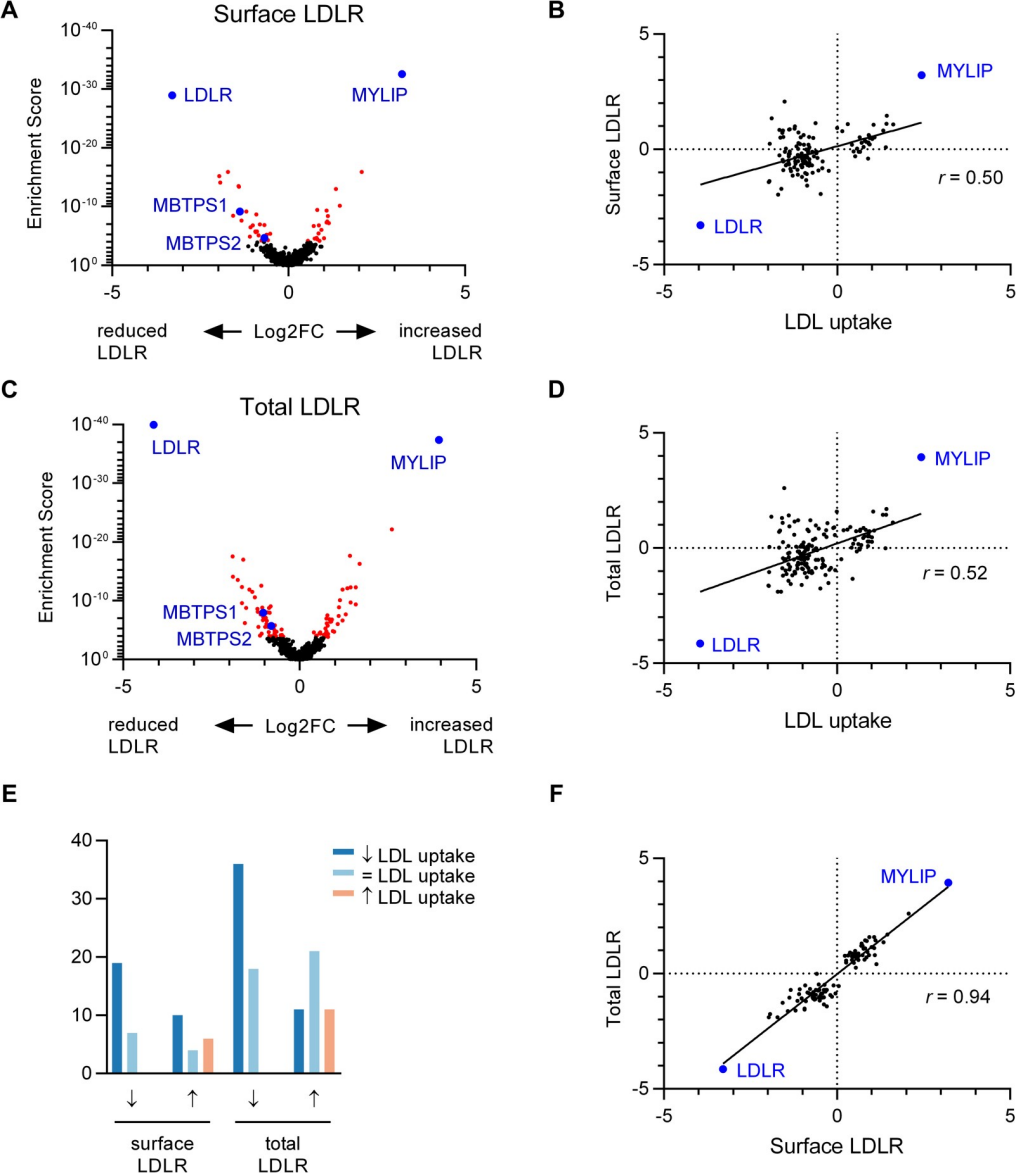
Orthogonal CRISPR screen for modifiers of LDLR abundance in HuH7 cells. (A) Volcano plot displaying surface LDLR abundance MAGeCK gene level enrichment score and log2 fold change for each gene tested in the customized gRNA library, with genes identified with FDR<5% displayed in red. (B) Comparison of effect size for LDL uptake and surface LDLR abundance for each gene showing a significant effect for either. (C) Volcano plot and (D) comparison of effect size for LDL uptake and total cellular LDLR abundance. (E) Comparison of corresponding effect on LDL uptake for each gene exhibiting an influence on surface or total LDLR abundance. (F) Comparison of effect size for each gene exhibiting an influence on either surface or total LDLR abundance.

To determine how each of our screen hits influences LDLR activity, we mutagenized HuH7 wild-type cells with our customized gRNA library and selected mutants by the amount of LDLR staining either at the cell surface (Fig 5A and 5B and S8 Table) or in semi-permeabilized cells (Fig 5C and 5D and S8 Table). As expected, the top hit associated with both decreased surface and decreased total LDLR was *LDLR* itself, and the top hit for increased surface and increased total LDLR was *MYLIP*. We identified 26 and 20 genes whose targeting either reduced or enhanced LDLR surface staining, respectively (FDR<0.05, Fig 5A). Screening for total LDLR similarly revealed 46 and 43 genes whose targeting either reduced or enhanced total cellular LDLR staining (FDR<0.05, Fig 5C). Most targeted genes exhibiting decreased LDLR staining (surface or total cell-associated) had also exhibited decreased fluorescent LDL uptake (Fig 5E). In contrast, gene targeted cells with increased surface or total LDLR exhibited heterogeneous effects on LDL uptake, with roughly equal numbers exhibiting either reduced or increased LDL uptake (Fig 5E). Targeted genes demonstrated a high degree of correlation between surface and total LDLR staining (Fig 5F), with no genes exhibiting significant effects on surface and total LDLR staining in opposite directions.

### Cell-type specificity of LDL uptake modifiers

**Fig 6:**
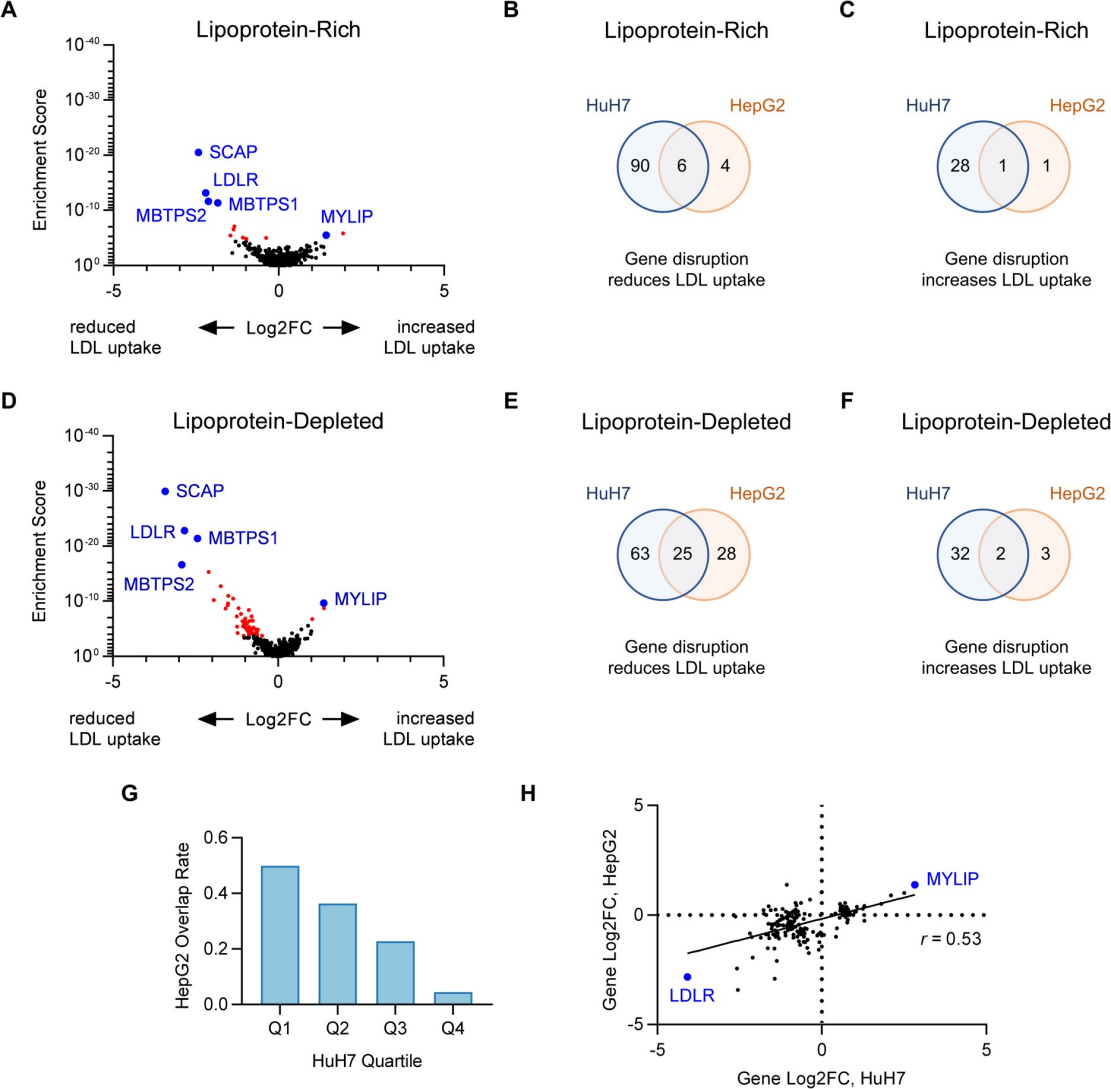
Orthogonal CRISPR screen for modifiers of LDL uptake by HepG2 cells. (A-F) Volcano plots displaying MAGeCK gene level enrichment score and log2 fold change for each gene tested in the secondary gRNA library, under lipoprotein-rich (A) or lipoprotein-depleted (D) cultured conditions, with genes identified with FDR<5% displayed in red. Venn diagrams demonstrating the overlap between HuH7 and HepG2 cells for positive (B, E) and negative (C, F) regulators of LDL uptake under lipoprotein-rich (B-C) or lipoprotein-depleted (E-F) culture conditions. (G) Positive regulators of LDL uptake in HuH7 cells under lipoprotein-depleted conditions were grouped by quartile and the proportion in each group that also influenced LDL uptake in HepG2 cells is displayed. (H) The effect size in lipoprotein-depleted conditions for gene-level gRNA enrichment in each cell type is plotted for genes showing a functional role in either cell type.

Comparison of our data to a previous siRNA screen for endothelial cell LDL uptake[19] revealed limited overlap, with only 1 gene identified in both studies (S9 Table). To examine whether the LDL uptake modifiers identified here might be unique to HuH7 cells, we also applied our customized library to a screen of LDL uptake in HepG2 cells. As in HuH7 cells, LDL uptake in HepG2 cells was dependent on *LDLR* and modulated by targeting of known regulators including *SCAP*, *MBTPS1*, *MBTPS2*, and *MYLIP* (Fig 6A and Fig 6D and S10 Table). Under lipoprotein-rich conditions, we identified only 10 and 2 genes whose targeting was associated with reduced or increased LDL uptake in HepG2 cells, respectively (Fig 6A), with 6/10 positive regulators (Fig 6B) and 1/2 negative regulators (Fig 6C) exhibiting similar effects in HuH7 cells. A much higher number of LDL uptake modifiers were identified under lipoprotein-depleted conditions, with disruption of 53 and 5 genes associated with reduced or increased LDL uptake, respectively (Fig 6D volcano). Among these latter genes, 25/53 positive regulators (Fig 6E) and 2/5 negative regulators (Fig 6F) exhibited similar effects in HuH7 cells. The likelihood of a gene showing a functional influence on LDL uptake by HepG2 cells was predicted by the strength of its association with LDL uptake by HuH7 cells (Fig 6G). Significant positive correlation was observed for the degree of enrichment for a given LDL uptake modifier in either cell line (Fig 6H). No genes were identified that associated with significant effects on LDL uptake in opposite directions in HuH7 and HepG2 cells.

### Opposing effects of exocyst disruption on HuH7 cellular LDL and transferrin uptake

**Fig 7:**
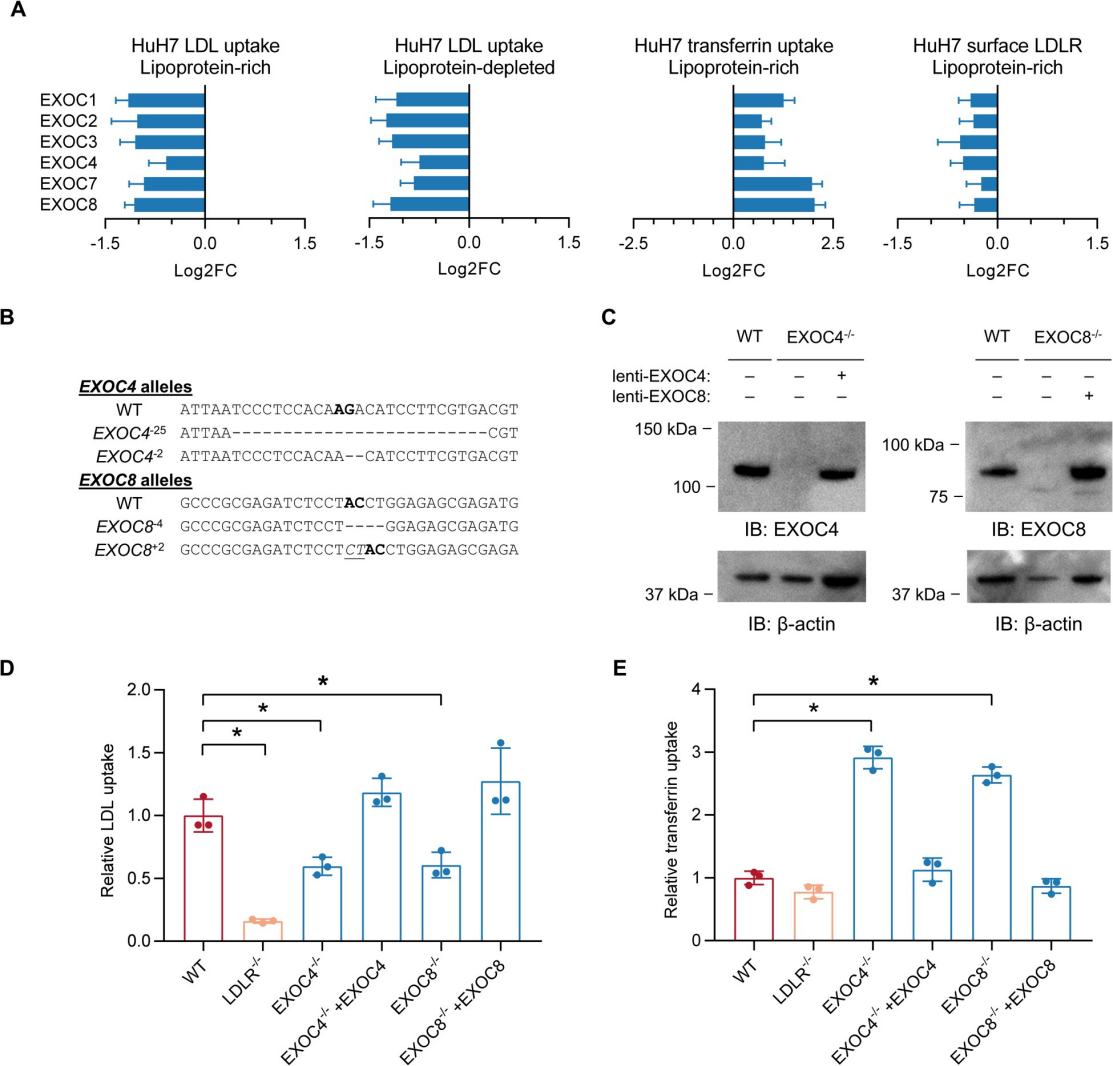
Disruption of the exocyst causes a discordant effect on HuH7 uptake of LDL and transferrin. (A) Average log2-fold change +/- SEM for the 15 gRNA in our secondary CRISPR library targeting each exocyst component in selected populations. Reduction in gRNA frequency reflects a decrease in LDL or transferrin uptake or LDLR staining. (B) Allele genotypes from individual HuH7 clones isolated after CRISPR targeting of either *EXOC4* or *EXOC8*. (C) Immunoblotting of lysates prepared from wild-type HuH7 cells, or *EXOC4*^*-25/-2*^ or *EXOC8*^*-4/+2*^ clones with and without ectopic lentiviral expression of a *EXOC4* or *EXOC8* CRISPR-resistant cDNA. (D) LDL uptake assay and (E) transferrin uptake assay of WT, *LDLR*^+1/+1^, and *EXOC4*^*-25/-2*^ or *EXOC8*^*-4/+2*^ clones with and without ectopic expression of a CRISPR-resistant cDNA. * = *p* < 0.05.

Among the candidate LDL uptake modifiers identified in our primary CRISPR screen were multiple components of the exocyst, an octameric protein complex involved in vesicular trafficking[20]. Disruption of each of the 6 exocyst genes targeted in the secondary CRISPR library was associated with a significant reduction (FDR<5%) of LDL uptake in HuH7 cells tested under either lipoprotein-rich or lipoprotein-depleted culture conditions (S2 Table). Intriguingly, each of these 6 exocyst genes was associated with an opposite effect on the uptake of transferrin (Fig 7A), suggestive of cargo selectivity rather than a global influence on endocytosis or receptor recycling. To further investigate this possibility, we generated individual HuH7 clones harboring biallelic frameshift indels in *EXOC4* or *EXOC8* (Fig 7B). To rule out an off-target effect as the cause of the phenotype, we also performed phenotypic rescue experiments with lentiviral expression constructs engineered with synonymous mutations that disrupt the CRISPR target site of each corresponding cDNA. Immunoblotting confirmed a loss of protein in each mutant cell line with restoration by ectopic expression of the CRISPR-resistant cDNA (Fig 7C). Mutant *EXOC4* and *EXOC8* clones each exhibited a ~40% decrease in LDL uptake and a ~2–3 fold increase in transferrin uptake, both of which were rescued by expression of the corresponding cDNA (Fig 7D and 7E).

## Discussion

Forward genetic screens are a powerful tool for the high-throughput and unbiased identification of genes that contribute to a biologic phenotype. Over the past decade, breakthroughs in genome editing technology have revolutionized the interrogation of gene function by improving the ease, speed, and accuracy of gene disruption. The programmability of CRISPR-mediated genome editing with a short gRNA sequence lends itself to large-scale oligonucleotide synthesis and quantification through massively parallel DNA sequencing. Together, these features make pooled CRISPR screening a powerful recent addition to the biologist’s toolkit.

We applied genome-wide CRISPR screening to identify novel determinants of cellular LDL uptake, identifying a large set of genes, many of which were not previously recognized to play a role in LDL uptake. The validity of our results is supported by several lines of evidence. First, we identified several well-established genes involved in cellular LDL uptake, with *LDLR* and *MYLIP* representing the top hits for positive and negative regulation of LDL uptake in both HuH7 and HepG2 cells, under both lipoprotein-rich and lipoprotein-depleted conditions, as well as for positive and negative regulation of cell surface and total LDLR abundance in HuH7 cells. Additional genes consistently identified across our screens included the positive control genes *SCAP*, *MBTPS1*, and *MBTPS2*. Second, our validation rate of hits was highly dependent on the strength of signal for a candidate gene in the primary screen, demonstrating a significant correlation over independent experiments. Third, our screen hits exhibited a high degree of concordance between lipoprotein-rich and lipoprotein-depleted conditions, much greater than might be expected by stochastic variation alone. This concordance also extended to the individual gRNA level, as gRNAs showing significant activity for one condition were much more likely to show activity for the other condition. Finally, we also observed a high degree of concordance between orthogonal screens. For example, genes whose perturbation impacted LDL uptake were much more likely to also be associated with reduced surface or total LDLR abundance.

Our findings highlight the value of following up candidate genes from a primary genome-wide CRISPR screen with a customized gRNA library. A more limited gene list allows for greater depth of gRNA per gene, infected cells per gRNA, and sequencing reads per gRNA. Reflecting these technical advantages, we observed significantly improved signal-to-noise ratio in our secondary screen, with more significant enrichment for positive hits, improved detection of internal positive controls, and no identification of negative control nontargeting genes. Generation of a secondary library also facilitates additional assays providing further biologic insight into screen hits, as we were more readily able to query candidate genes under different selective pressures.

Despite these strengths, a number of caveats apply to our screen data. First, our screen was performed in immortalized hepatoma cells, removed from the *in vivo* environment and evaluated in two-dimensional cell culture. While it is reassuring that this system recapitulates the LDLR-dependent, SREBP-responsive nature of cellular LDL uptake, the extent to which these interactions extend to the physiologic setting remains uncertain. The high degree of cell type specificity for our screen hits emphasizes the need for empirical testing of identified genes in other contexts. Second, our tracer for LDL uptake was prepared by NHS ester labeling of amines in the proteinaceous component of purified LDL particles. While the LDLR-dependence of uptake for this tracer argues against a disruption of the ligand-receptor interaction, it is possible that the molecular dependencies of native LDL uptake may differ. Third, the threshold for determining what constitutes a valid result is somewhat arbitrary. It is likely that among our list of hits are a subset of false positives, and likewise that among our genes which did not pass validation are a number of false negatives. Fourth, our screen may not uncover genes truly involved in LDL uptake if those genes also are either essential or confer a fitness advantage in culture, since gRNAs targeting those genes will be progressively depleted from the pooled population over the duration of the experiment. Fifth, our screen is unable to detect genes that perform redundant functions in LDL uptake, as compensation may prevent a significant phenotypic effect. For example, despite their clear roles in LDLR expression, we did not detect significant effects upon disruption of *SREBF1* or *SREBF2*, likely due to overlapping functions allowing one gene to compensate for loss of the other[7]. Finally, our screen is limited in detecting only those genes which exhibit a phenotype through a cell-autonomous effect. For example, PCSK9 induces LDLR degradation after its secretion. Therefore, *PCSK9*-targeted cells are still susceptible to the activity of PCSK9 secreted by neighboring cells, preventing these mutants from developing alterations in LDLR abundance and LDL uptake.

Orthogonal testing of our customized gRNA library provided us with initial insight into the mechanism of effect for each of our screen hits. Disruption of most LDL uptake regulators did not cause a similar reduction in transferrin uptake and for some genes instead caused opposing effects. This latter group includes all 6 tested subunits of the exocyst, an octameric protein complex that was originally identified for its role in vesicular trafficking in budding yeast[20,21]. The exocyst is recruited to vesicle membranes by small GTPases and mediates the tethering of these vesicles to target membranes. A prior study found that intracellular injection of an antibody against Sec8 (encoded by *EXOC4*) into polarized MDCK cells disrupted LDLR trafficking[22]. Our findings provide further genetic support for a role of the exocyst in LDLR regulation by HuH7 cells. The molecular mechanism by which the exocyst promotes LDL uptake is unclear but seems likely to affect the kinetics of LDLR recycling. Receptor recycling from endosomes is a complex process that involves distinct pathways involving different adaptors, protein sorting machinery, and membrane composition[23–25]. LDL and transferrin initially enter into common endosomes via clathrin-mediated endocytosis but then quickly segregate[26–28]. Prior investigations have implicated the retriever, CCC, and WASH complexes in LDLR recycling[29,30], and we did detect some of these components (Vps29, CCDC22) as positive regulators of LDL uptake in HuH7 cells. Our findings are consistent with a model in which the exocyst is preferentially recruited to and promotes the recycling of LDLR-containing vesicles.

Screening of LDL uptake in a *LDLR*-deleted clone confirmed that many of our screen hits were dependent on *LDLR* for their functional influence on LDL uptake. Several LDL uptake modifiers however also seemed to influence LDL uptake on a *LDLR*-deleted genetic background. These included *SCAP*, *MBTPS1*, and *MBTPS2*, suggesting that the SREBP axis regulates additional mediators of LDL endocytosis beyond LDLR, consistent with clinical findings that statins reduce LDL cholesterol and mortality in LDLR null patients with homozygous familial hypercholesterolemia[31]. Similarly, disruption of the exocyst was also associated with reduced LDL uptake on a *LDLR*-deleted background. The molecular basis for residual LDL uptake in *LDLR*-deleted cells is not well understood, but may be mediated by alternative receptors for LDL. For example, *SCARB1* encodes a scavenger receptor that binds a variety of ligands including LDL, has SREBP-binding sites in its promoter region, and is expressed in hepatocytes[32]. Supporting this model, we found disruption of *SCARB1* to be associated with reduced LDL uptake in both WT and *LDLR*-deleted HuH7 cells. LDL uptake regulators may therefore still contribute to LDL uptake on a *LDLR*-deleted background if they also influence *SCARB1* or other pathways mediating this residual LDL uptake. The presence of an LDLR-independent effect for a LDL uptake regulator does not rule out a concurrent LDLR-dependent mechanism. For example, the ~40% reduction in LDL uptake resulting from exocyst disruption in WT cells is unlikely to be fully explained by an LDLR-independent effect, given that LDL uptake in *LDLR*-deleted cells is only ~15% of that in WT cells.

We found that many LDL uptake regulators did not exhibit a readily detectable impact on LDLR levels either at the cell surface or associated with the entire cell, despite the apparent specificity of this antibody-based detection, with *LDLR* and *MYLIP* representing the top positive and negative regulators for each screen. The basis for this discrepancy is unclear but may be related either to screen hits influencing LDLR kinetics or function rather than steady state levels, or to compensatory effects in mutant cells that upregulate *LDLR* expression in response to defective LDL uptake.

Functional annotations of our novel screen hits showed modest enrichment in some pathways, including N-glycosylation, ubiquitination, and transcriptional regulation. In addition, the regions containing the identified genes were enriched for significant associations with LDL in a genome-wide association study of nearly 400,000 Europeans. Our findings provide further support for the involvement of these genes in human cholesterol regulation and suggest a molecular mechanism for their involvement in human lipid traits.

In summary, we identified a list of high-confidence genetic modifiers of HuH7 cell LDL uptake, with supporting evidence for their specificity, mechanism of action, and generalizability. These findings highlight the power of genome-scale CRISPR screening and offer new avenues for understanding the molecular determinants of cellular LDL uptake.

## Materials and methods

### Reagents

HuH7 cells were cultured in DMEM containing 10% FBS and penicillin/streptomycin. Cellular uptake assays were performed with fluorescent conjugates of LDL (Cayman Chemical, Ann Arbor MI, 10011229) or transferrin (ThermoFisher Scientific, Waltham MA, T35352). For immunoblotting, membranes were probed with antibodies against LDLR (Abcam, Cambridge UK, ab52818, 1:2000), β-actin (Santa Cruz Biotechnology, Dallas TX, sc-47778, 1:5000), EXOC4 (Abcam, ab205945, 1:1000), and EXOC8 (Santa Cruz, sc-515532, 1:500). For flow cytometry, cells were stained with a fluorescently-conjugated antibody against LDLR (R&D Systems, Minneapolois MN, FAB2148G). CRISPR-mediated gene disruption was performed by cloning gRNA sequences into BsmBI sites of pLentiCRISPRv2 (Addgene #52961, a gift from Feng Zhang[17]) or into BbsI sites of pX459 (Addgene #62988, a gift from Feng Zhang). Genotyping was performed by Sanger sequencing of PCR amplicons of genomic target sites with individual alleles deconvoluted by TIDE analysis of chromatograms[33]. Lentiviral expression constructs were generated by assembly of cDNA sequences (GE Healthcare Dharmacon, Lafayette CO, EXOC4 MHS6278-202759993 and EXOC8 MHS6278-202758964) and a blasticidin resistance cassette into the LeGO-ic2 plasmid (Addgene # 27345, a gift from Boris Fehse[34]) using HiFi DNA Assembly Master Mix (NEB, Ipswich MA).

**Table:** 

**Oligonucleotide Sequences**	
***LDLR***	
gRNA	AACAAGTTCAAGTGTCACAG
Target PCR forward	TCCCAAAGTGCTGGGATTAC
Target PCR reverse	GGCAGAGTGGAGTTCCCAAA
Sanger	TCCCAAAGTGCTGGGATTAC
***EXOC4***	
gRNA	ACGTCACGAAGGATGTCTTG
Target PCR forward	ACCTAGGAAAAAGAGCACGCTGTA
Target PCR reverse	CGCCCCCATACGGTGACCAG
Sanger	ACCTAGGAAAAAGAGCACGCTGTA
***EXOC8***	
gRNA	GGCCCGCGAGATCTCCTACC
Target PCR forward	TGAGGCGCGGCTGTACGTGA
Target PCR reverse	CAGGCTGCTTTTCTGCTCGGTC
Sanger	TGAGGCGCGGCTGTACGTGA
***Non-targeting***	
gRNA	CGTGTGTGGGTAAACGGAAA

### Primary screen of HuH7 cellular LDL uptake

For each biologic replicate, 62.5 million HuH7 cells were harvested and evenly distributed into 12 separate 15 cm^2^ tissue culture plates. Pooled lentivirus containing the GeCKOv2 library[17] was added to cells in suspension at an estimated MOI of 0.4. The following day puromycin was added at a concentration of 1 μg/mL to select for infected cells. Cultured cells were then harvested, pooled, and passaged as needed to maintain logarithmic phase growth. Total cell numbers were maintained above 25 million cells (representing 200X coverage of the gRNA library) throughout the entirety of the screen. On assay day 12, cells were split into duplicate plates. On day 13, cells were either maintained in lipoprotein-rich media, or the media exchanged to DMEM supplemented with 10% lipoprotein-depleted fetal calf serum (Sigma S5394). On day 14, plates were sequentially processed by aspiration of media, washing in PBS, and addition of serum-free DMEM containing 4 μg/mL DyLight549-conjugated human LDL (Cayman Chemical, Ann Arbor MI, 10011229). Cells were incubated for 1 hr at 37°C then harvested with trypLE express, centrifuged 500x*g* for 5 min, washed in PBS, centrifuged again, resuspended at 20 million cells/mL PBS, and filtered into FACS tubes. Cell suspensions were then analyzed on a BD FACSAria III with cells exhibiting the top and bottom 7.5% DyLight^549^ fluorescence sorted into separate collection tubes. Genomic DNA was isolated using a DNEasy DNA isolation kit (Qiagen, Hilden, Germany). Preparation of barcoded amplicon libraries and mapping and deconvolution of sequencing reads obtained from an Illumina NextSeq sequencing run were performed as previously described[14]. Enrichment analysis was performed using the MAGeCK software package[35].

### Design and synthesis of secondary CRISPR library

Candidate genes from the primary LDL uptake screen were sorted by their relative ranking for MAGeCK gene level enrichment score. An FDR cutoff of 50% and 75% was used to select candidate positive and negative regulators, respectively. The candidate gene list was entered into the Broad Genetic Perturbation Platform sgRNA Designer for selection of 15 optimized targeting sequences per gene[36]. Nontargeting controls, long non-coding RNA, and microRNA candidates for which a corresponding target sequence could not be readily identified in the GPP platform were omitted. A total of 12 additional genes serving as internal controls (e.g. *TFRC* for transferrin uptake) or hypothesis-driven candidates (e.g. *SREBF2* for LDL uptake) were manually added to the candidate gene lists. Flanking sequences were appended to gRNA sequences to serve as priming sites for PCR amplification. Synthesized pooled oligonucleotides were obtained from CustomArray (Bothell, WA), amplified 18 cycles with Herculase II DNA polymerase (Agilent, Santa Clara CA), and purified using a QIAquick PCR purification kit (Qiagen). Assembly was performed with 1650 ng of BsmBI-digested pLentiCRISPRv2 and 250 ng of amplicon in a total reaction volume of 100 μL with HiFI DNA Assembly Mix (NEB) for 30 min at 50°C. Assembly products were purified with a QIAquick PCR purification kit, electroporated in triplicate into Endura electrocompetent cells (Lucigen, Middleton WI), and plated onto 24.5 cm^2^ LB-agar plates. After 14 hr at 37°C, bacteria were harvested and plasmid DNA purified with an EndoFree Plasmid Maxi kit (Qiagen). Dilution plates of electroporated cells confirmed a colony count of >100X relative to the size of the gRNA library. Library diversity was assessed with a Illumina MiSeq run of gRNA amplicons prepared as previously described[14].

### Validation and orthogonal screening of LDL uptake modifiers

Lentiviral infection with the customized CRISPR library, selection of infected cells, passaging, and parameters for LDL uptake were performed as in the primary genome-wide CRISPR screen. Transferrin uptake was performed with 5 μg/mL AlexaFluor^555^-conjugated transferrin (ThermoFisher) in serum-free DMEM for 30 min at 37°C. LDLR staining was performed for 30 min on ice with a 1:50 dilution of AlexaFluor^488^-conjugated LDLR antibody (R&D Systems, Minneapolois MN, FAB2148G) into PBS supplemented with 1% FBS, with or without 0.1% Tween-20 for surface or total cellular staining, respectively. Treated cells were sorted into high and low populations of fluorescence, genomic DNA isolated, and gRNA sequencing performed as in the primary screen. Three replicates were performed for each screen. Cell numbers were maintained above a minimum depth of coverage of 500X relative to the customized gRNA library throughout the screen until the time of sorting. For each sort, approximately 10–20 million cells were analyzed with 1–2 million cells collected per population.

### Generation of CRISPR-targeted HuH7 clones

HuH7 cells were transfected with a *LDLR*-targeting CRISPR pX459 construct using Lipofectamine LTX (ThermoFisher) or transduced with lentivirus prepared from *EXOC4* or *EXOC8*-targeting lentiCRISPRv2 constructs. After puromycin selection of transfected cells was complete, serial dilutions of cells were plated into 96 well plates. Wells containing a single colony of growth were then selected for expansion. Single cell clones were analyzed by Sanger sequencing and TIDE deconvolution of PCR amplicons at the CRISPR target site, and by immunoblotting with antibodies against LDLR, EXOC4, EXOC8, and β-actin.

### Comparison to GWAS lipid trait associations

Association analysis for LDL cholesterol was performed using SAIGE[37] for 388,629 individuals in the white British subset of UK Biobank[38]. Inverse-normalized residuals for LDL after adjustment for batch, principle components 1–4, age, and age^2 were generated separately in males and females and then combined. Pre-treatment LDL levels were estimated for individuals on lipid-lowering medication by dividing the measured LDL value by 0.7. Control genes for comparison with the experimentally identified genes were selected based on nearest matching for both total gene length and total exon length. Gene transcription and exon start and end positions were taken from the refFlat file provided by the USCS genome annotation database[39]. Genes that overlapped within 500 kb of the identified gene start and end positions were excluded from the pool of control genes prior to matching. Significance for the difference in distribution of GWAS result p-values between the identified genes and selected control genes was determined using a two-sided Kolmogorov-Smirnov test.

### Functional annotation of LDL uptake modifiers

A total of 163 genes for which targeting in the secondary CRISPR screen was associated with either an increase or decrease in LDL uptake with FDR<5%, under either lipoprotein-rich or lipoprotein-depleted conditions, were included for analysis. This gene list was queried for enrichment of Gene Ontology classifications relative to all genes in the reference human genome using the PANTHER statistical overrepresentation test (PANTHER version 15.0, release February 14, 2020)[40]. Complete results are given in S4 Table. Classifications with p-value <10^−4^ at the most terminal node in the hierarchy for each subgroup are displayed in S6 Fig.

## Supporting information

S1 Fig Development of conditions for primary screen of cellular LDL uptake. (A) Flow cytometry of HuH7 cells incubated for 1 hr in serum-free media with 4 μg/mL DyLight549-conjugated LDL, compared to autofluorescence of untreated HuH7 cells. (B) Dose-response curve of fluorescent signal acquisition by HuH7 cells incubated with a range of concentrations of DyLight549-conjugated LDL. (C) Time course of uptake of 4 μg/mL DyLight549-conjugated LDL by HuH7 cells. (D) Relative uptake was quantified by flow cytometry for WT HuH7 cells and cells targeted by CRISPR with a LDLR-targeting gRNA, or a nontargeting control gRNA, in cells that were pre-treated for 24 with lipoprotein-depleted media or maintained in lipoprotein-rich media.
(TIF)

S2 Fig Concordance between HuH7 LDL uptake primary screen hits. The number of genes identified as positive regulators (FDR<0.05) under lipoprotein-rich and/or lipoprotein-depleted culture conditions is displayed.
(TIF)

S3 Fig Synthesis of a customized gRNA library targeting candidate HuH7 LDL uptake regulators. (A) The number of unique gRNA sequences among the starting pooled oligonucleotide template and the synthesized plasmid pool are shown. (B) The number of mapped sequencing reads for each gRNA as a function of its relative rank in representation among all gRNAs. The ratio of reads for the gRNA at the 90^th^ and 10^th^ percentiles of representation are shown.
(TIF)

S4 Fig Strategy for secondary CRISPR validation screen and orthogonal screens. Mutagenesis of HuH7 WT cells, HuH7 LDLR KO cells, or HepG2 cells with the customized gRNA library is performed and pooled populations of mutants undergo selection by flow cytometry on the basis of relative LDL uptake, transferrin uptake, surface LDLR staining, or total cellular LDLR staining. The frequency of each gRNA in cells with high or low fluorescence is assessed by massively parallel DNA sequencing of gRNA amplicons, with computational analysis performed using the MAGeCK algorithm.
(TIF)

S5 Fig Concordance between HuH7 LDL uptake secondary screen hits. (A) For the secondary screen of HuH7 LDL uptake, the number of genes identified with a FDR<0.05 or FDR>0.05 under lipoprotein-rich and lipoprotein-depleted culture conditions is displayed. (B) Correlation between the degree of enrichment under lipoprotein-rich or lipoprotein-depleted conditions for each of the 15 gRNA for every target gene validated under both conditions by gene-level analysis.
(TIF)

S6 Fig Functional annotations of validated LDL uptake regulators. Genes whose disruption was associated with a significant increase or decrease in LDL uptake, under either lipoprotein-rich or lipoprotein-depleted conditions, were analyzed by Gene Ontology classifications. Annotations demonstrating an enrichment with p<10^−4^ are displayed. Parental classifications for each are omitted from this figure and included in S3 Table.
(TIF)

S7 Fig Influence of culture conditions on LDLR-independent LDL uptake modifiers. (A-B) Venn diagrams of genes whose disruption either reduced (A) or (B) enhanced LDL uptake under lipoprotein-rich or lipoprotein-depleted culture conditions. (C) Gene Ontology analysis was performed for the 79 genes whose disruption reduced LDL uptake in LDLR-deleted cells under either condition. The most terminal annotations in a hierarchy demonstrating an enrichment with p<10^−4^ are displayed.
(TIF)

S1 Table MAGeCK analysis of primary genome-wide CRISPR screen of HuH7 LDL uptake in lipoprotein-rich culture conditions. (XLSX)

S2 Table MAGeCK analysis of primary genome-wide CRISPR screen of HuH7 LDL uptake in lipoprotein-depleted culture conditions. (XLSX)

S3 Table MAGeCK analysis of targeted secondary CRISPR screens for modifiers of LDL uptake by HuH7 cells. (XLSX)

S4 Table Functional annotation of HuH7 LDL uptake regulators identified in this study. (XLSX)

S5 Table Significant UK Biobank LDL GWAS results within and nearby each gene. (XLSX)

S6 Table MAGeCK analysis of orthogonal CRISPR screen for modifiers of transferrin uptake by HuH7 cells. (XLSX)

S7 Table MAGeCK analysis of orthogonal CRISPR screen for modifiers of LDL uptake by LDLR-deleted HuH7 cells. (XLSX)

S8 Table MAGeCK analysis of orthogonal CRISPR screen for modifiers of LDLR abundance in HuH7 cells. (XLSX)

S9 Table Comparison of genes identified in a previous siRNA screen of LDL uptake by endothelial cells and in this screen. (XLSX)

S10 Table MAGeCK analysis of orthogonal CRISPR screen for modifiers of LDL uptake by HepG2 cells. (XLSX)
