# Supplementary figures and images for: Genome-scale CRISPR screening for modifiers of cellular LDL uptake
Source: PLoS Genet. 2021 Jan 29;17(1):e1009285. doi: 10.1371/journal.pgen.1009285 (PMC7875399; doi:10.1371/journal.pgen.1009285)

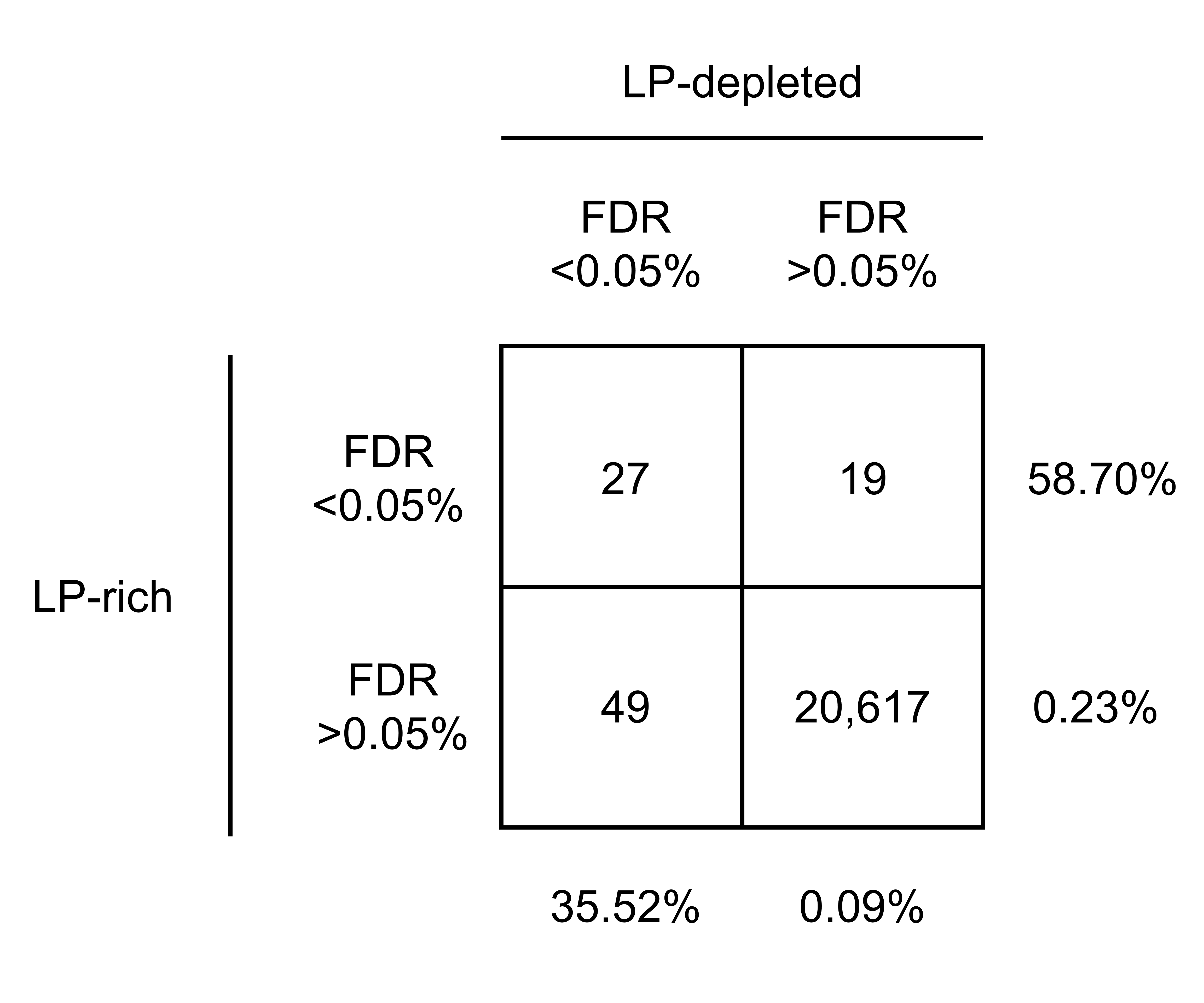

Supplement: S2 Fig — The number of genes identified as positive regulators (FDR<0.05) under lipoprotein-rich and/or lipoprotein-depleted culture conditions is displayed. (TIF) [file pgen.1009285.s002.tif]
